# Plant Hormones in Phytoplasma Infected Plants

**DOI:** 10.3389/fpls.2019.00477

**Published:** 2019-04-17

**Authors:** Marina Dermastia

**Affiliations:** Department of Biotechnology and Systems Biology, National Institute of Biology, Ljubljana, Slovenia

**Keywords:** hormone crosstalk, host plant, jasmonic acid, phytoplasma, plant hormone, salicylic acid

## Abstract

Phytoplasmas are bacterial plant pathogens that need a plant host and an insect vector for their spread and survival. In plants, the physiological responses that phytoplasmas trigger result in symptom development through effects on hormonal, nutritional, and stress signaling pathways, and the interactions between these. In this review, recent advances on the involvement of plant hormones together with their known and deduced roles in plants infected with phytoplasmas are discussed. Several studies have directly, or in many cases indirectly, addressed plant hormone systems in phytoplasma-infected plants. These have provided accumulating evidence that phytoplasmas extensively affect plant hormone pathways. Phytoplasmas thus, with disturbing complex plant hormone networks, suppress plant immunity and modify plant structure, while optimizing their nutrient acquisition and facilitating their colonization of the plants, and their dissemination among plants by their insect vectors.

## Introduction

Phytoplasmas are cell wall-less bacteria belonging to the class Mollicutes (Bertaccini and Lee, 2018). Analysis of their genomes suggests that they have developed from Gram-positive bacteria by a process of reductive evolution. Consequently, they are very small in size, they have the smallest genome of any known plant bacteria and they have limited metabolic pathways (Kube et al., 2008, 2012; Oshima et al., 2013; Marcone, 2014). Phytoplasmas thrive and multiply in both plant and hemipteran insect hosts. The wild reservoirs of phytoplasmas, e.g., common alder (*Alnus glutinosa*) (Maixner and Reinert, 1999), elm (*Ulmus* spp.) (Katanić et al., 2016; Mehle et al., 2017), or clematis (*Clematis vitalba*) (Angelini et al., 2004) may exhibit typical symptoms of phytoplasma diseases, but often they remain free from obvious symptoms. However, under specific environmental conditions and with specific insect vectors, phytoplasmas can change the plant host and cause disease. Therefore, several phytoplasmas have been discovered to be pathogens of economically important crops (Bertaccini et al., 2014; Dermastia et al., 2017). With the particular economic importance of phytoplasma diseases, most of available data have been collected from infected crops and plants with a horticultural value, and not by more common ways that use model plants to study their interactions with microbes.

In plants, phytoplasmas exclusively inhabit the phloem tissues. As they have an obligatory parasitic lifestyle, it is still not possible to grow them routinely under *in vitro* conditions (Contaldo et al., 2012, 2016). Consequently, our knowledge of their biology is fragmented, and in most cases it remains indirect, as deduced from the known genome sequences of a few phytoplasmas (Shigeyuki and Yasuko, 2015) and/or from transcriptomic, proteomic, and metabolomic studies of the diseased plants (Ehya et al., 2013; Abbà et al., 2014; Gai et al., 2014b, 2018b; Liu et al., 2014; Luge et al., 2014; Fan et al., 2015a,b,c, 2017; Mardi et al., 2015; Nejat et al., 2015; Dong et al., 2016; Dermastia, 2017; Snyman et al., 2017; Wang et al., 2018).

Initially phytoplasma diseases were classified as auxonic, which means affecting the plant host growth (Horsfall and Dimond, 2012). Now it becomes evident that the pathogenicity of phytoplasmas involves a number of effector proteins, which have profound and diverse effects not only on growth, but also on other aspects of plant life. Phytoplasmas secret their effectors into the cytoplasm of the phloem sieve cells (Bai et al., 2009; Hoshi et al., 2009; MacLean et al., 2011, 2014; Sugio et al., 2011b; Anabestani et al., 2017; Kitazawa et al., 2017). In the cells where they finally unload from the phloem, these effector proteins trigger physiological responses in the plant that result in symptom development. Infected plants can show different symptoms, including proliferation of shoots (i.e., witches' broom), stunting, yellowing, formation of leaf-like tissues instead of flowers (i.e., phyllody), and greening of the floral organs (i.e., virescence). Such physiological conditions appear to be a consequence of phytoplasma effects on hormonal, nutritional, and both, developmental and stress signaling pathways, and the interactions between these.

It has already been shown that most phytoplasma-induced symptoms mirror alterations in gene expression and/or changed protein levels in infected plants, as compared to uninfected plants. Several of these changes are related to genes associated with the expression of hormones. Plant hormones are naturally occurring, small, organic molecules that have important regulatory roles in plant growth and developmental processes (Rijavec and Dermastia, 2010; Wasternack and Song, 2016; Liu et al., 2017; Ahanger et al., 2018; Binenbaum et al., 2018; Camara et al., 2018; Ma et al., 2018; Skalický et al., 2018; Weijers et al., 2018; Wasternack and Strnad, 2019). They are also involved in plant defense responses against pathogens (Pieterse et al., 2012; Shigenaga and Argueso, 2016; Shigenaga et al., 2017). Plant hormones act through complex networks of interactions, referred to as the hormone crosstalk, which results in changes to the plant physiology, including plant immunity (Kohli et al., 2013). The key plant hormones involved in plant immunity are salicylic acid (SA), jasmonic acid (JA) and ethylene. Other hormones, however less studied, are also important parts of plant defense signaling pathways, such as abscisic acid, auxins, gibberellic acid, cytokinins, brassinosteroids, and peptide hormones (Bari and Jones, 2009; Shigenaga and Argueso, 2016; Shigenaga et al., 2017). Their roles appear to be in the fine tuning of responses to pathogens through activation of the specific physiological processes that are required to defend a plant against invading organisms (Shigenaga and Argueso, 2016).

Due to several limitations associated with phytoplasma research, few studies have directly addressed the levels of plant hormones in phytoplasma-infected plants (e.g., Lazar, 2010; Sugio et al., 2011a, 2014; Gai et al., 2014b; Luge et al., 2014; Minato et al., 2014; Paolacci et al., 2017). However, there are several indirect indications of the involvement of plant hormones in phytoplasma–plant interactions. Of note, the studies that suggest the plant hormone contribution to pathogenesis are very diverse. The analyses were performed on different parts of plants, e.g., whole leaves (Gai et al., 2014a; Youssef et al., 2018), leaf midribs (Hren et al., 2009; Prezelj et al., 2016a), phloem sap (Gai et al., 2018a); they use different approaches, e.g., direct hormone evaluation (Lazar, 2010) or gene expression analyses (Hren et al., 2009; Musetti et al., 2013; Paolacci et al., 2017); and very different techniques, e.g., HPLC analysis (Youssef et al., 2018), microarray data or real-time PCR analyses (Hren et al., 2009), or RNA-seq data analysis (Snyman et al., 2017). Taking this into account, the results of these studies are not directly comparable.

Here, the involvement of plant hormones together with their known and deduced roles in plants infected with phytoplasmas (Table 1) are reviewed and discussed.

**Table 1:** Summarized studies on phytoplasma infected plants, which report plant hormones.

**Hormone**	**Phytoplasma**	**Host plant**	**Up (↑)/down (↓) regulation of** ***genes*****/proteins involved in biosynthesis and/or signaling**		**Hormone level**	**Suggested role**	**References**
Salicylic acid	‘*Ca*. P. solani’	Grapevine (*Vitis vinifera*)	↑	*VvPR1.1/1*.2; *VvPR2*; *VvBGL2*; *VvGlc2, VvOlp*; *TL4*; *TL5*; *VvTHAU2*; *VvOsm*; *VvSamt*; *VvNPR1*.2; *VvPAL1-8*; *VvPAL9; VvPAL11*; *VvICS*	↑	SAR, partly antagonizing the expression of some JA-responsive genes	Hren et al., 2009; Landi and Romanazzi, 2011; Santi et al., 2013; Dermastia et al., 2015; Prezelj et al., 2016b; Paolacci et al., 2017; Rotter et al., 2018
			↑/↓	*VvPAL1-8*;	↑		
	‘*Ca*. P. solani’ (PO)	Tomato (*Solanum lycopersicum)*	↑	*ICS*; *PR1a*; *PR1b*; *PR2a*; *PR4*; *PR5*; *PR7B*; *PR1*			Ahmad et al., 2013
			↓	*PAL*			
	‘*Ca*. P. solani’ (C)	Tomato (*Solanum lycopersicum)*	↑	*PAL*; *ICS*; *PR1a*; *PR1b*; *PR2a*; *PR4*; *PR5*; *PR7*; *PR10*			Ahmad et al., 2013
	Flavescence dorée phytoplasma	Grapevine (*Vitis vinifera*)	↑ ↓	*PR-1; BGL/*BGL*; THAU/*THAU *NPR1; PR-10*	↑	SAR	Margaria and Palmano, 2011; Gambino et al., 2013; Margaria et al., 2013; Prezelj et al., 2016a
	‘*Ca*. P. asteris’ (AY-OY, AY-SY, AY-SW1, AY-SW2, AY-LY1, AY-LY2)	Garland chrysanthemum (*Chrysanthemum coronarium*)	↑	*TLP*			Zhong and Shen, 2004
	‘*Ca*. P. asteris’ (AY-WB)	SAP11- *Arabidopsis* plants	↑	*PR-1*		Altered root architecture	Lu et al., 2014
	Peanut witches'-broom phytoplasma	Madagascar periwinkle (*Catharanthus roseus*)	↑ ↓	*CrPR1.1*; *CrPR1.2* *CrSID2*			Liu et al., 2014
	‘*Ca*. P. aurantifolia’	Mexican lime (*Citrus aurantifolia*)	↑	SA-related genes			Mardi et al., 2015
	‘*Ca*. P. mali’	Apple (*Malus domestica*)	↑	*MdPR-1*; *MdPR-2*; *MdPR-5*	↑/↓		Musetti et al., 2013; Janik et al., 2017
	‘*Ca*. P. mali’	Apple (*Malus domestica*) plantlets	↓	*PR-1a*; *PR-2*			Giorno et al., 2013
	Yellow decline phytoplasma	Coconut (*Cocos nucifera*)	↑	*TL5*; *Glc2*			Nejat et al., 2015
Jasmonic acid	‘*Ca*. P. solani’	Grapevine (*Vitis vinifera*)	↑	*VvLox1*; *VvLox2*; *VvLox4*; *VvAOS2*; *VvAOS5*; *VvAOS7*; *VvAOC1*; *VvAOC2; VvOPR1*; *VvJMT2*; *VvJMT3/4*; *VvMYC2*; *VvJAZ1, VvJAZ2, VvJAZ3*; *VvCHIT III*	↑		Hren et al., 2009; Dermastia et al., 2015; Paolacci et al., 2017; Rotter et al., 2018
			↑/↓	*VvPR4*; *VvPIN*;			
			↓	*VvPR10.1/10.3*			
	‘Ca. P. solani’ (PO)	Tomato (*Solanum lycopersicum)*	↓	*PIN2; PIN2b; LOXD*			Ahmad et al., 2013
	‘Ca. P. solani’ (C)	Tomato (*Solanum lycopersicum)*	↑	*PIN2; PIN2b; LOXD*			Ahmad et al., 2013
	Flavescence dorée phytoplasma	Grapevine (*Vitis vinifera*)	↓	*OPR; VvJAZ3; VvLOXA; VvOPR3*			Gambino et al., 2013; Chitarra et al., 2018
	‘*Ca*. P. asteris’ (AY-OY)	Arabidopsis (*Arabidopsis thaliana*)	↓	*LOX2*			Minato et al., 2014
		TENGU- *Arabidopsis* plants	↓	*LOX2*	↓	Impaired flower maturation, sterility	Minato et al., 2014
	‘*Ca*. P. asteris’ (AY-WB)	SAP11- *Arabidopsis* plants	↓	*LOX2*		Alluring insects	Sugio et al., 2011a; Lu et al., 2014
	‘*Ca*. P. aurantifolia’	Mexican lime (*Citrus aurantifolia*)	↑/↓	JA-related genes			Monavarfeshani et al., 2013; Mardi et al., 2015
	‘*Ca*. P. mali’	Tobacco (*Nicotiana occidentalis*)	↑	*AOS; ECH; ACAA*			Luge et al., 2014
	‘*Ca*. P. mali’	Apple (*Malus domestica*)	↓	*MdAOS2*; *Md12-OPR3*; *MdPI II*	↑ /↓		Musetti et al., 2013; Janik et al., 2017
	Paulownia witches' broom phytoplasma	*Paulownia fortunei*	↑	*HEC2*; *PLD*			Fan et al., 2015c
	Jujube witches' broom phytoplasma	Chinese jujube (*Ziziphus jujuba**)***	↑	*23 kDa jasmonate-induced-like protein*	↑		Ye et al., 2017
Ethylene	‘*Ca*. P. solani’	Grapevine (*Vitis vinifera*)	↑	*VvEtr*			Hren et al., 2009
	Flavescence dorée phytoplasma	Grapevine (*Vitis vinifera*)	↑	*ACO*			Gambino et al., 2013
	‘*Ca*. P. aurantifolia’	Mexican lime (*Citrus aurantifolia*)	↑	*AP2-like ethylene responsive transcription factor*; *ethylene-induced esterase*			Monavarfeshani et al., 2013; Mardi et al., 2015
			↑/↓	Ethylene related genes; *ethylene-responsive transcription factor*			
	Paulownia witches' broom phytoplasma	*Paulownia fortunei*	↑	*ERF1*; *RAP2.3*; *ACC synthase*; *ACC oxidase*; *pyk1*			Fan et al., 2015c
	Yellow decline phytoplasma	Coconut (*Cocos nucifera*)	↑	*ERF114*; *ACO*			Nejat et al., 2015
Auxin	Flavescence dorée phytoplasma	Grapevine (*Vitis vinifera*)	↓	*VvARF*			Chitarra et al., 2018
	‘*Ca*. P. asteris’ (AY-OY)	Arabidopsis *(Arabidopsis thaliana)*	↓	*ARF6; ARF8*			Minato et al., 2014
		TENGU- Arabidopsis plants	↓	*ARF6*; *ARF8*; *AUX/IAA*; *SAUR*; *GH3*; auxin efflux carrier family proteins	↓	Impaired flower maturation, sterility	Hoshi et al., 2009; Minato et al., 2014
	‘*Ca*. P. aurantifolia’	Mexican lime (*Citrus aurantifolia*)	↑ ↑/↓	*AUX/IAA protein* *Auxin induced protein;* auxin related genes	↑		Ehya et al., 2013; Monavarfeshani et al., 2013; Mardi et al., 2015
	Yellow decline phytoplasma	Coconut (*Cocos nucifera*)	↑	*Auxin-induced protein 5NG4*			Nejat et al., 2015
	Jujube witches' broom phytoplasma	Chinese jujube (*Ziziphus jujuba**)***	↓	*Auxin-responsive protein*	↓		Ye et al., 2017
Cytokinins	‘*Ca*. P. solani’	Grapevine (*Vitis vinifera*)	↑	*VvHP*			Hren et al., 2009
	Paulownia witches' broom phytoplasma	*Paulownia fortunei*	↑ ↓	*IPT; cisZOG* *CYP735A*			Fan et al., 2014
	Yellow decline phytoplasma	Coconut (*Cocos nucifera*)	↑ ↓	*IPT* *CKX*			Nejat et al., 2015
	Coconut lethal yellowing phytoplasma	Coconut (*Cocos nucifera*)			↓		Aguilar et al., 2009
	Phytoplasma causing branching phenotype	*Euphorbia pulcherrima*	↑	*AHP1*		Branching phenotype	Nicolaisen and Horvath, 2008
	Not determined	Madagascar periwinkle (*Catharantus roseus*)			↑	Virescent flowers	Davey et al., 1981
	Mulberry yellow dwarf phytoplasma	Mulberry (*Morus multicaulis*)	↑ ↓	*IPT* *CKX*	↑		Gai et al., 2014a
	Phytoplasmas: Faba bean phyllody; Brinjal little leaf; Strawberry green petal	Madagascar periwinkle (*Catharantus roseus*)			↑/↓		Lazar, 2010
Abscisic acid	‘*Ca*. P. aurantifolia’	Mexican lime (*Citrus aurantifolia*)	↑/↓	ABA related genes			Mardi et al., 2015
	‘*Ca*. P. mali’	Apple (*Malus domestica*)			↑		Janik et al., 2017
	Paulownia witches' broom phytoplasma	*Paulownia fortunei*	↑	*PYL*; *ABF*			Fan et al., 2014
	Mulberry yellow dwarf phytoplasma	Mulberry (*Morus multicaulis*)	↑	*zeaxanthin epoxidase*; *9-cis-epoxycarotenoid dioxygenase*	↑		Gai et al., 2014a
Gibberellins	‘*Ca*. P. aurantifolia’	Mexican lime (*Citrus aurantifolia*)	↑	*CPS; KS; KO; KAO; GA3OX; GA2oxs; lolO2; casbene synthase*			Mardi et al., 2015
	Peanut witches'-broom phytoplasma	Madagascar periwinkle (*Catharanthus roseus*)	↓	*CrGA1.1*			Liu et al., 2014
	Yellow decline phytoplasma	Coconut (*Cocos nucifera*)	↑	*GA2oxs*		Stunting, inflorescence necrosis and premature nut fall	Nejat et al., 2015
	Phytoplasma causing branching phenotype	*Euphorbia pulcherrima*	↑	*GAST-1-like*		Branching phenotype	Nicolaisen and Horvath, 2008
Brassinosteroids	‘*Ca*. P. aurantifolia’	Mexican lime (*Citrus aurantifolia*)	↓	BA related genes			Ehya et al., 2013; Mardi et al., 2015

*Gene names are taken from the original studies*.

## Salicylic Acid

In plants, the phenolic hormone SA exists as both a free acid and in conjugated forms that result from its glucosylation, hydroxylation, and methylation (Lee et al., 1995). The major conjugate is usually SA 2-*O*-glucopyranosyl (syn. SA 2-O-β-D-glucoside, https://pubchem.ncbi.nlm.nih.gov/compound/151505#section=Synonyms). SA was the first of the plant hormones for which a role in plant defense against pathogens was shown (White, 1979). It is involved in local defense systems against biotrophic and hemibiotrophic pathogens, as well as in systemic acquired resistance (Fu and Dong, 2013). NON-EXPRESSOR OF PATHOGENESIS RELATED PROTEIN 1 (NPR1) and its paralogs, NPR3, and NPR4, have been proposed as SA receptors (Fu et al., 2012; Wu et al., 2012). NPR1 regulates gene expression by physically interacting with TGA transcription factors. These bind to the promoters of the *PATHOGENESIS RELATED* (*PR*) *PROTEIN* genes, the expression of which is thus activated in the presence of SA (Fu and Dong, 2013). Among the several *PR* genes that encode PR proteins (van Loon et al., 2006), PR-1, PR-2, and PR-5 are induced by SA and are commonly used as molecular markers for the SA-dependent systemic acquired resistance signaling (Frías et al., 2013; Fu and Dong, 2013).

### Effects of Phytoplasma Infection on Salicylic Acid Biosynthesis and Signaling

Salicylic acid studies have revealed that several salicylate biosynthetic genes are up-regulated in the midribs or whole leaves of grapevine infected with the phytoplasma ‘*Ca*. P. solani’, e.g., *VvICS*, which encodes isochorismate synthase, and *VvSamt*, which encodes S-adenosyl-L-methionine: salicylic acid carboxyl methyltransferase, which catalyzes the formation of the volatile ester methyl salicylate from SA (Hren et al., 2009; Dermastia et al., 2015; Paolacci et al., 2017; Rotter et al., 2018). In addition, quantification of SA and its conjugates in the main leaf veins during infections of grapevine with ‘*Ca*. P. solani’ showed a 26-fold increase in SA 2-*O*-glucopyranosyl (Prezelj et al., 2016b), and significantly higher free and total SA, as compared to uninfected grapevine (Paolacci et al., 2017). Salicylic acid-glucopyranoside and salicylate are also greatly increased in the grapevine main leaf veins infected with the flavescence dorée phytoplasma (Prezelj et al., 2016a).

The relative expression of *VvNPR1* is not different across grapevine infected with ‘*Ca*. P. solani’, as compared to uninfected plants or plants recovered from this infection. However, a small up-regulation of *VvNPR1.2* in symptomatic leaves was only reported for one growing season. In contrast, the levels of the transcript of another gene involved in SA signaling, *VvEDS1*, were significantly higher in symptomatic infected grapevine, as compared to uninfected plants. This same study showed that some salicylate responsive genes from the WRKY transcription family are up-regulated in leaves of symptomatic grapevine and of grapevine recovered from the disease bois noir, which is caused by the ‘*Ca*. P. solani’ phytoplasma (Paolacci et al., 2017).

### The Salicylic Acid Marker PR-Proteins

More data are available on the expression of genes that encode the SA-signaling marker PR proteins. These are up-regulated in phytoplasma-infected seedlings of garland chrysanthemum (Zhong and Shen, 2004) and in leaf vascular tissues of Mexican lime infected with ‘*Ca*. P. aurantifolia’ (Monavarfeshani et al., 2013). Increased expression of the gene that encodes the PR-1 protein was shown for mulberry phloem sap infected with phytoplasmas (Gai et al., 2018a).

In *Catharanthus roseus* flowers infected with peanut witches'-broom phytoplasma, a transcriptome profile revealed up-regulation of the *CrPR1.1* and *CrPR1.2* genes (Liu et al., 2014). In grapevine infected with ‘*Ca*. P. solani’, expression of *VvPR1.1/1.2* was increased in leaves as compared to uninfected plants (Paolacci et al., 2017). In addition, increased levels of the leaf transcripts of *VvGLC2, VvGLC1*, and *VvGLC3* have been shown to be involved in the development of bois noir in grapevine (Hren et al., 2009; Landi and Romanazzi, 2011; Dermastia et al., 2015; Paolacci et al., 2017). These genes encode an isoform of β-1,3-glucanases, a member of the class 2 PR proteins (PR-2). In addition, their expression (Gambino et al., 2013) together with high levels of the β-1,3-glucanase protein product have been demonstrated in the grapevine leaves infected with the flavescence dorée phytoplasma (Gambino et al., 2013). There is also an increase in the leaf transcript levels of genes from the *PR-5* class upon infection with ‘*Ca*. P. solani’ (Hren et al., 2009; Santi et al., 2013; Dermastia et al., 2015; Paolacci et al., 2017; Rotter et al., 2018) and for the phytoplasma that causes flavescence dorée (Margaria and Palmano, 2011; Gambino et al., 2013; Margaria et al., 2013; Prezelj et al., 2016a). Of further interest, an association between *PR-5* gene expression and susceptibility of a grapevine cultivars has been reported, whereby *PR-5* leaf transcript levels were higher in less susceptible grapevine cultivars (Margaria and Palmano, 2011; Prezelj et al., 2016a).

Increased expression of *PR-1a, PR-2a* and *PR-5* as compared to uninfected plants was shown in leaves after infection of tomato with the ‘*Ca*. P. solani’ strains PO and C (Ahmad et al., 2013), and for apple tree infected with ‘*Ca*. P. mali’ (Musetti et al., 2013). Expression of *PR-5* and *PR-2* were also increased in coconut leaves infected with yellow decline phytoplasma (Nejat et al., 2015).

For mulberry phloem sap infected with phytoplasmas, induced expression of *MuMLPL329* was seen, which encodes major latex protein-like 329. Ectopic expression of this gene enhances mulberry resistance to several pathogens, including phytoplasmas. Although treatment of wild-type mulberry seedlings with SA did not affect *MuMLPL329* expression, the *PR-2* and *PR-5* genes were highly expressed in *MuMLPL329*-overexpressing transgenic *Arabidopsis* plants (Gai et al., 2018a).

Of note, in contrast to field grown plants, in *in vitro* micro-propagated apple plantlets infected with ‘*Ca*. P. mali’, the relative expression levels of *PR-1a* and *PR-2* were lower than in uninfected plants, while expression of the gene that encodes the thaumatin-like protein from the PR-5 group was not changed. On the other hand, up-regulation of *PR-6* and *PR-8* was observed in these infected apple plantlets (Giorno et al., 2013).

### The *DMR6* Susceptibility Gene

The *DMR6* (*downy mildew resistance 6*) gene encodes an oxidoreductase (2-oxoglutarate (2OG)-Fe(II) oxygenase) (Van Damme et al., 2005, 2008; Zeilmaker et al., 2015), and this is required for susceptibility of *Arabidopsis* to *Hyaloperonospora arabidopsidis*, a causal agent of downy mildew in *Arabidopsis* (Van Damme et al., 2008). The DMR6 protein has recently been functionally characterized as SA 5-hydroxylase, which catalyzes the formation of 2,5-dihydroxybenzoic acid in *Arabidopsis*. This has essential roles in mediation of SA homeostasis during plant development, leaf senescence, and pathogen responses (Zhang et al., 2017). It has been suggested that *DMR6* acts as a susceptibility *S* gene in a class of suppressors of plant immunity (Zeilmaker et al., 2015). The expression levels of a grapevine ortholog of *DMR6* that shows 69% similarity to the sequence of the *AtDMR6* gene from *Arabidopsis* was higher in grapevine plants that were infected with ‘*Ca*. P. solani’ and flavescence dorée phytoplasma, as compared to uninfected plants (Prezelj et al., 2016a; Rotter et al., 2018).

### The Exogenous Application of SA-Analog

A treatment of grapevine cuttings exposed to the infectious vector of flavescence dorée phytoplasma *Scaphoideus titanus* with a functional analog of SA, acibenzolar-S-methyl, reduced the rate of infected plants, but was ineffective in inducing recovery from disease under field conditions (Miliordos et al., 2017).

## Jasmonic acid

Jasmonic acid and its derivatives are lipid-derived signaling compounds that are formed through the lipoxygenase pathway from the α-linolenic acid of chloroplast membranes. JA is a major plant hormone that is involved in stress responses, such as insect attack, wounding, drought, and attack by necrotrophic pathogens (Yan and Xie, 2015). JA is also a signal in several developmental processes (Wasternack, 2015; Wasternack and Song, 2016). In addition, recent studies have shown that some microbes avoid host plant defense systems to promote host susceptibility through hijacking of the JA pathway (Yan and Xie, 2015).

### Effects of Phytoplasma Infection on Jasmonic Acid Biosynthesis and Signaling

In grapevine leaves infected with ‘*Ca*. P. solani’, most jasmonate biosynthetic genes show up-regulation, as compared to uninfected plants (Paolacci et al., 2017). Specifically, among the four *VvLOX* genes that encode lipoxygenases, the transcripts of two were significantly higher in symptomatic and non-symptomatic leaves, as well as in the leaves of grapevine recovered from bois noir. The other two *VvLOX* genes were significantly up-regulated only in the leaves of non-symptomatic and recovered plants. In contrast, in other studies with the same grapevine cultivar infected with ‘*Ca*. P. solani’, the *VvLOX* genes were significantly up-regulated in symptomatic leaves (Hren et al., 2009; Dermastia et al., 2015; Rotter et al., 2018), but not in leaves of the recovered plants (Dermastia et al., 2015). In this pathosystem, it has been shown that expression of several genes that encode allene oxide synthase and allene oxide cyclase involved in JA biosynthesis (Wasternack and Strnad, 2019) depends on the period of the growing season, as well as the disease status (Paolacci et al., 2017). As compared to uninfected plants, the *VvOPR1* gene, which encodes 12- oxo-phytodienoic acid reductase, and the *VvJMT2* and *VvJMT3/4* genes, which encode jasmonate-O-methyltransferase, are up-regulated in leaves of infected and recovered plants. In agreement with the increased expression of *VvJMT2* and *VvJMT3/4* in these plants, the levels of methyl-jasmonate were also significantly higher. In the JA signaling pathway, the gene that encodes transcription factor MYC2 was up-regulated, as also seen for some of the genes that encode the jasmonate ZIM-domain genes proteins (JAZ). In addition, it has been shown that some of the genes that encode jasmonate-inducible WRKY transcription factors are slightly down-regulated in leaves of symptomatic plants, as compared to those of recovered and uninfected plants.

The genes that encode allene oxide synthase 2, oxophytodienoate reductase and JA-inducible proteinase inhibitor II were up-regulated in apple tree leaves following recovery from infection with ‘*Ca*. P. mali’, as compared to diseased plants (Musetti et al., 2013). Tobacco infected with the ‘*Ca*. P. mali’ strain AT showed up-regulation of α-linolenic acid metabolism in leaf veins, which is consistent with increased JA levels (Luge et al., 2014). On the contrary, in leaves of Mexican lime infected with ‘*Ca*. P. aurantifolia’ the *LOX1* and *AOS* genes, and the genes that encode the JAZ protein that inhibits the expression of JA-responsive genes, were down-regulated. However, *JMT* gene expression was increased in response to phytoplasma infection (Mardi et al., 2015).

Transcriptomic analysis of mulberry phloem sap infected with phytoplasmas showed differential expression of several mRNAs associated with JA metabolism and signaling (Gai et al., 2018a). In leaves of Chinese jujube in an earlier stage after infection of phytoplasma causing jujube witches' broom, there were large changes in a JA-induced protein-like gene transcript. Associated with this, the JA levels were increased in the early stages of infection. However, later on, up-regulation of this gene decreased, and that of the gene that encodes the linoleate 13S-lipoxygenase 2-like protein showed a more than 6-fold decrease (Ye et al., 2017).

In transgenic *Arabidopsis* plants that constitutively expressed the phytoplasma virulence effector peptide TENGU, which was first identified in the ‘*Ca*. P. asteris’ strain AY-OY and is known to induce dwarfism (Hoshi et al., 2009), JA levels were decreased (Minato et al., 2014). *Arabidopsis* plants infected with the ‘*Ca*. P. asteris’ strain AY-WB in general produce less JA in young leaves as compared to old leaves and uninfected plants (Sugio et al., 2011a). Additionally, it was shown in the same study that binding of the phytoplasma effector SAP11 destabilized the CINCINNATA-related TEOSINTE BRANCHED1/CYCLOIDEA/PROLIFERATING CELL FACTORS (CIN-TCP) 1 and 2 transcription factors, which control plant development and promote expression of the *LOX* genes. Consequently, SAP11-mediated destabilization of CIN-TCP resulted in down-regulation of *LOX* genes and JA synthesis, and an increase in a progeny of the AY-WB insect vectors. In apple tree during an infection with ‘*Ca*. P. mali’, the binding of TCP transcription factors to the SAP11-like effector ATP_00189 was associated with higher levels of 7-*iso*-jasmonyl-*L*-isoleucine and *cis*-12-oxo-phytodienoic acid in spring, when the phytoplasmas were not found in the canopy. Their accumulation was not induced further up to the end of the growing season. On the other hand, in uninfected trees the levels of both of these compounds increased from spring to fall. Therefore, in fall the concentration of JA acid-related compounds were lower in infected plants compared to uninfected ones (Janik et al., 2017). A study on grapevine infected with flavescence dorée phytoplasma showed a decrease in *VvTCP* transcript levels, which in leaf midribs occurs in parallel with reductions in *VvLOXA* and *VvOPR3* expression (Chitarra et al., 2018).

### Jasmonic Acid-Responsive Genes

While JA-responsive *PR3* and *PR4* were not affected in leaves of symptomatic grapevine infected with ‘*Ca*. P. solani’ and *PR6* was repressed, these genes were up-regulated in plants recovered from bois noir (Paolacci et al., 2017). An up-regulation of the PR-6 gene *PIN2* was seen in leaves of tomato plants infected with ‘*Ca*. P. solani’ strain C, although this gene was down-regulated upon infection with strain PO (Ahmad et al., 2013). Up-regulation of the genes that encode PR-3 and PR-10 was shown after infection of coconut with yellow decline phytoplasma (Nejat et al., 2015).

In *Paulownia fortunei* infected with paulownia witches' broom phytoplasma, there was an indirect indication of increased JA biosynthesis and JA signal transduction, as seen by up-regulation of genes that encode phospholipase D and transcription factor HEC2 in leaves (Fan et al., 2015c).

### The Exyogenous Application of Jasmonic Acid

The expression of the *major latex-protein-like* gene (*MuMLPL329*) was increased in the mulberry phloem sap infected with phytoplasmas and after spraying of mulberry leaves with JA solution, suggesting an involvement of JA in pathogenesis. Of note, similar application of SA did not have any effect on the *MuMLPL329* expression (Gai et al., 2018a).

## Ethylene

Ethylene is a small hydrocarbon gas hormone in plants that controls the development of leaves, flowers, and fruits (Iqbal et al., 2017). In addition, ethylene is considered to be an important component of the plant defense against pathogens (Shigenaga and Argueso, 2016). Ethylene signaling is often in synergy with JA signaling, and it activates expression of some defense genes, which results in increased resistance to pathogens (Glazebrook, 2005; Shigenaga and Argueso, 2016).

Significant differential expression of genes related to ethylene signaling has been reported for Mexican lime infected with ‘*Ca*. P. aurantifolia’, including the genes that encode APETALA2/Ethylene (AP2/ERF)-like-responsive transcription factor, ethylene-responsive transcription factor, and ethylene-induced esterase (Mardi et al., 2015). Similarly, several genes involved in synthesis, degradation, and regulation of ethylene were significantly differentially expressed in leaf midribs of uninfected grapevine and of those infected with ‘*Ca*. P. solani’ (Hren et al., 2009). The gene that encodes the last enzyme in ethylene biosynthesis, 1-aminocyclopropane-1-carboxylate oxidase, was overexpressed in leaf midribs during the yellow decline phytoplasma-coconut interaction (Nejat et al., 2015). In mulberry infected with phytoplasmas, the micro RNA mul-miR2111a-5p was predicted to target this gene (Gai et al., 2014a). This gene was significantly up-regulated in leaves in the response of *Paulownia fortunei* to infection with paulownia witches' broom phytoplasma together with genes that encode the ethylene receptor, 1-aminocyclopropane-1-carboxylate synthase, and AP2/ERF (Fan et al., 2015c). In addition, *AP2/ERF*–like mRNA was identified as a possible target of miR172 in grapevine infected with ‘*Ca*. P. asteris’ (Snyman et al., 2017), Chinese jujube infected with witches'-broom phytoplasma (Shao et al., 2016), and in phytoplasma infected mulberry (Gai et al., 2014a). It was suggested that activated regulatory pathway of miR156-SQUAMOSA PROMOTER BINDING PROTEIN LIKE-miR172 may lead to abnormal leaf shape, downward curling of leaves, flower abortion and sterility, and phyllody (Shao et al., 2016; Snyman et al., 2017).

## “There are Many Magic Rings in This World…and None of Them Should be Used Lightly” (J. R. R. Tolkien, The Lord of the Rings)

### Auxins

Indole-3-acetic acid (IAA) is the most abundant and basic auxin native to plant functions. IAA generates the majority of the auxin effects in intact plants, such as stimulation of growth and in responses to gravity or light stimuli. In addition, elevated IAA levels or auxin signaling can also promote disease development in some plant–pathogen interactions (Kunkel and Harper, 2018; Weijers et al., 2018).

An example where IAA levels were significantly increased, as compared to healthy trees, were leaves of phytoplasma-infected Mexican lime (Ehya et al., 2013). On the other hand, in the same pathosystem, two genes encode IAA-amido synthetase, which prevents free IAA accumulation, and these were significantly up-regulated (Mardi et al., 2015). In the early stages after infection of Chinese jujube with jujube witches' broom phytoplasma, several genes involved in auxin production and signaling were down-regulated, and the auxin content decreased by some 2.5-fold (Ye et al., 2017).

Infection of Mexican lime with ‘*Ca*. P. aurantifolia’ was characterized by induced expression of genes that encode some auxin-responsive proteins from the large SAUR family of proteins, and with increased transcript levels of a gene that encodes an AUX/IAA protein in leaves (Mardi et al., 2015). This protein family is involved in suppression of auxin-responsive genes, through binding to and inactivation of auxin response factors. These are positive regulators of the auxin signaling pathway (Robert-Seilaniantz et al., 2011), and have also been shown to be differentially expressed in Mexican lime upon infection with ‘*Ca*. P. aurantifolia’ (Mardi et al., 2015).

The phytoplasma virulence factor TENGU represses transcription of auxin efflux-related and auxin-responsive genes, including *auxin response factors ARF6* and *ARF8*, which results in lower auxin levels and growth inhibition of apical buds in TENGU-transgenic *Arabidopsis* (Hoshi et al., 2009; Minato et al., 2014).

#### Exogenous Applications of Auxins

For shoots of *C. roseus* grown *in vitro*, treatment with IAA and another auxin, indole-3-butryc acid, induced recovery from phytoplasma infection. While in recovered plants, ‘*Ca*. P. pruni’ and ‘*Ca*. P. asteris' were not detected, or were detected only in a second nested PCR, ‘*Ca*. P. solani' instead persisted in plants with induced remission of the symptoms (Curković, 2008). It has been suggested that the pathway that leads to hormone-dependent remission of the symptoms differs from that for natural recovery (Leljak-Levanić et al., 2010). Tomato plants infected with Columbia Basin PPT phytoplasma and treated with IAA showed partially restored healthy profiles of certain PPT-responsive proteins suggesting that these proteins may be involved in hormone homeostasis (Ding et al., 2011). A study in which *C. roseus* was sprayed with an auxin concluded that the auxin-promoted resistance is JA independent (Tai et al., 2013).

### Cytokinins

Cytokinins are a major group of plant hormones that are involved in the control of various processes during plant growth and development. Chemically, they are *N*^6^-substituated adenine derivatives, which include their respective ribotides, ribosides, and glucosides. Interconversions between different cytokinin metabolites represent the transition between active, inactive, storage, and transport forms, and *in vivo* this process is relatively dynamic and rapid (Rijavec and Dermastia, 2010). Although some symptoms of phytoplasma-infected plants hint at participation of cytokinins, such as with witches' broom, leaf yellowing, fasciations, reports of their involvement in phytoplasma–plant interactions are scarce and mainly indirect.

It has been suggested that the virescent flowers of phytoplasma-infected *C. roseus* plants are the result of increased levels of cytokinins in flowers (Davey et al., 1981). In *C. roseus* infected with phytoplasmas that cause faba bean phyllody, brinjal little leaf, and strawberry green petal, increases in biologically active cytokinin ribosides and decreases in the irreversibly inactive end products of cytokinin metabolism, 9-glucosides, were observed, as compared to uninfected plants. However, these increases were more pronounced in infected leaves than infected roots, stems, and flowers. Of note, no differences were seen for the total cytokinin levels between infected and uninfected plants (Lazar, 2010). In mulberry infected with phytoplasmas that cause mulberry yellow dwarf disease, the cytokinin biosynthetic gene encoding isopentenyl transferase was up-regulated in the infected leaves, while the cytokinin biodegradation gene encoding cytokinin oxidase was down-regulated. It was suggested that this might result in higher concentrations of zeatin, zeatin riboside, and isopentenyladenosine in infected leaves and phloem sap compared to those in uninfected ones, and consequently a broken plant hormone balance in the infected mulberry plants (Gai et al., 2014a). In coconut palm infected with coconut lethal yellowing, there were symptoms of lethal yellowing, and the content of free bases, ribosides and 9-glucosides of isopentenyl adenine-type cytokinins were drastically decreased, as compared to uninfected controls (Aguilar et al., 2009).

The infection of coconut with yellow decline phytoplasma resulted in down-regulation of the expression of the gene that encodes cytokinin dehydrogenase in leaf midribs (Nejat et al., 2015). Similarly, the transcript levels of the gene *ICYP735A* that encodes cytokinin *trans*-hydroxylase were lower than for uninfected leaves for *Paulownia fortunei* infected with Paulownia witches' broom phytoplasma (Fan et al., 2014).

Up-regulation of the *Ep11* gene was detected in the ornamental plant *Euphorbia pulcherrima* when infected with phytoplasmas. It has been suggested that Ep11 has a role in a desirable branching phenotype through the loss of apical dominance (Nicolaisen and Horvath, 2008). Ep11 has high similarity to a histidine-containing phosphotransmitter from *Arabidopsis* that is known to have a key role in the phospho-relay signal transduction pathway from cytokinin histidine kinase receptors to transcriptional response regulators in the nucleus (Sheen, 2002). An ortholog of *Ep11* was differentially expressed in grapevine leaf midribs infected with ‘*Ca*. P. solani' (Hren et al., 2009; Rotter et al., 2018).

### Abscisic Acid

Abscisic acid is a sesquiterpene signaling molecule with an important regulatory role in plant growth, and in seed development and dormancy, as well as in plant–microbe interactions (Lievens et al., 2017).

The concentration of abscisic acid in the leaves of coconut palms infected with coconut lethal yellowing phytoplasma increased during development of lethal yellowing (León et al., 1996). In mulberry infected with phytoplasmas that cause mulberry yellow dwarf disease the *zeaxanthin epoxidase* and *9-cis-epoxycarotenoid dioxygenase* encoding the key enzymes in the synthesis of abscisic acid were up-regulated, while the *abscisic acid 8*′*-hydroxylase* involved in degradation of abscisic acid was down-regulated in the infected leaves and phloem sap compared to the uninfected plants. These findings were in agreement with the higher level of abscisic acid in the infected plants (Gai et al., 2014a).

A transcriptome analysis of Chinese jujube leaves and flowers infected with jujube witches' broom phytoplasmas revealed down-regulation of the gene than encodes zeaxanthin epoxidase (Wei et al., 2017). During infection of *Paulownia fortune* with Paulownia witches' broom phytoplasma, the expression of the genes that encode the abscisic acid receptor PYL and the abscisic acid responsive element binding factor ABF were up-regulated in leaves (Fan et al., 2014). In *C. roseus* plants infected with two strains of ‘*Ca*. P. fraxini' there was no association between a decrease of stomatal conductance and the levels of abscisic acid as the main messenger to environmental stresses that promote stomatal closure. Therefore, an alternative mechanism whereby sucrose accumulation in the apoplast of guard cells leads to stomatal closure was proposed (Tan et al., 2001).

### Gibberellins and Brassinosteroids

The gibberellins comprise a large group of diterpenoid carboxylic acids that are ubiquitous in higher plants. Some gibberellins function as plant hormones, to promote organ expansion and developmental changes (Hedden and Thomas, 2012). On the other hand, brassinosteroids are plant polyhydroxylated steroidal hormones that have crucial roles in plant growth, development, and immunity (Tang et al., 2016). Growing evidence has indicated that brassinosteroids regulate the biosynthesis of gibberellins in rice (Tong et al., 2014) and in *Arabidopsis* (Unterholzner et al., 2015).

Transcriptional profiling of coconut infected with the yellow decline phytoplasma revealed up-regulation of the gene that encodes gibberelin-2-oxidase, which appeared to result in decreased gibberellin levels and development of symptoms of stunting, inflorescence necrosis, and premature nut fall (Nejat et al., 2015). Significant increases in the transcript levels of several genes involved in gibberellin biosynthesis, and significant down-regulation of genes that encode the gibberellin receptors GID1 and the DELLA protein RGL2, were reported in leaves during infection of Mexican lime with ‘*Ca*. P. aurantifolia’ (Mardi et al., 2015). In the same study, several genes related to biosynthesis and signaling of brassinosteroids were up-regulated.

## Hormone Crosstalk

There is increasing evidence that to govern plant immunity, plant hormones act interdependently, through complex antagonistic and synergistic interactions. This hormone crosstalk is primarily a consequence of transcriptional and translational co-regulation, and the outcomes of this complex hormone network are changes in plant physiology that result in defense responses against pathogens. However, frequently there is a trade-off between growth and defense. Our current understanding of these interactions is that growth and immunity are tightly co-regulated, and many negative correlations between growth and defense are the result of regulatory crosstalk (Karasov et al., 2017). On the other hand, successful pathogens have developed sophisticated molecular mechanisms to interfere with hormone biosynthesis and/or hormonal signaling pathways (Pieterse et al., 2012).

### “One Ring to Rule Them All” (J. R. R. Tolkien, the Lord of the Rings)

The “master ring” of the plant hormones involved in plant immunity consists of SA, JA, and ethylene (Bari and Jones, 2009; Shigenaga and Argueso, 2016), and tight co-regulation among these hormones is largely conserved across plant species (Karasov et al., 2017). SA is generally involved in activation of defense responses against biotrophic and hemi-biotrophic pathogens, while JA and ethylene are usually associated with defense against necrotrophic pathogens and herbivorous insects. In spite of these mutually antagonistic interactions, there is also evidence of synergistic interactions (van Wees et al., 2000; Mur et al., 2006; Tsuda et al., 2009; Liu et al., 2016; Zhang et al., 2018). It has also been demonstrated recently that during effector-triggered immunity SA and JA both accumulate to high level. Here, the early induction of JA-responsive genes and *de-novo* synthesis of JA that follows SA accumulation is activated through the SA receptors, which promote degradation of the JAZ JA transcriptional repressors (Liu et al., 2016). In addition, multiple hormone mutant analysis suggested that plants have a more robust immune system in which all three of these main hormones contribute to defense. However, in response to a particular pathogen and its style of infection, one hormone sector can have a greater contribution than the others (Tsuda et al., 2009). It seems that participation of other hormones and regulation of the crosstalk between different hormonal pathways during the infection depend on specific host plant-phytoplasma pathosystem (Table 1), as has been previously suggested for any plant-pathogen interaction (Bari and Jones, 2009).

Evidence collected in this review shows that an up-regulation of SA-signaling is involved in most studied interactions between phytoplasmas and host plants (Table 1). It has been suggested, therefore, that the observed increase in SA-signaling might be associated with a systemic acquired resistance (Ahmad et al., 2015; Dermastia et al., 2015; Prezelj et al., 2016a; Rotter et al., 2018). However, there is no indication that activation of the SA-signaling results in conferring resistance against phytoplasma infection.

Growing evidence suggests an ethylene-auxin crosstalk in which growth responses to ethylene are largely dependent on auxin (Zemlyanskaya et al., 2018). Although ethylene-auxin imbalance was observed in lethal-yellowing affected coconut palms in an early study of mycoplasma-like organisms (León et al., 1996), an in-depth analysis of ethylene-auxin crosstalk has not been a part of recent studies in phytoplasma-plant interactions.

### Phytoplasma Effector Proteins and Hormone Crosstalk

Pathogens have adopted innovative strategies to manipulate plant defenses that are regulated by hormones. The tactics that have been frequently used by plant pathogens involve disruption of hormone signaling pathways, for example by producing hormones and/or crosstalk via pathogen-derived effectors (Sugio et al., 2011b; Kazan and Lyons, 2014; Kunkel and Harper, 2018). Since phytoplasmas do not have genes for biosynthesis of plant hormones as it is evident from known whole or draft phytoplasma genome sequences, a secretion of effectors is thought to be a mechanism by which phytoplasma affect plant development.

Phytoplasma effectors can be identified by their cleavable signal peptides that are associated with a functional Sec-dependent phytoplasma pathway (MacLean et al., 2011). Transgenic *Arabidopsis* plants with stably expressed *SAP11*, which encode SAP11 of ‘*Ca*. P. asteris’ strain AY-WB, showed suppressed JA synthesis, and JA and SA defense responses (Sugio et al., 2011a; Lu et al., 2014; Tan et al., 2016). It has been shown that SAP11 reduces JA synthesis by destabilizing the class II TCP transcription factors (Sugio et al., 2011a, 2014). The same mode of action has been demonstrated for a SAP11-like effector from ‘*Ca*. P. mali’ during infection of apple tree and it was suggested that SAP11-like proteins might be key players in phytoplasmal infection (Janik et al., 2017).

SA-mediated responses are activated in grapevine infected with the flavescence dorée phytoplasma (Gambino et al., 2013; Prezelj et al., 2016a), with concurrent repression of JA (Gambino et al., 2013; Chitarra et al., 2018) and *VvARF* (Chitarra et al., 2018). The results here suggested that the JA decrease might involve a similar mechanisms to that shown for SAP11, with involvement of *VvTCP* (Chitarra et al., 2018). Of note, these responses were not enough for induction of resistance against the flavescence dorée phytoplasma. Although the pathogenesis in grapevine infected with the flavescence dorée phytoplasma or ‘*Ca*. P. solani’ is very similar in several aspects (Hren et al., 2009; Prezelj et al., 2016a,b; Rotter et al., 2018), it appears that these two differ in these basic defense responses. After infection of grapevine with ‘*Ca*. P. solani’, a synergistic increase in expression of genes associated with SA and JA biosynthesis and metabolism has been shown, as well as for SA and JA metabolites (Hren et al., 2009; Dermastia et al., 2015; Paolacci et al., 2017; Rotter et al., 2018). However, at the moment there are some indications that effectors of ‘*Ca*. P. solani’ other than SAP11-like are involved in this interaction (Čepin, 2018), which might induce different plant responses to this phytoplasma. Although the mode of action of different effectors from different strains of ‘*Ca*. P. solani’ is not known at the moment, it has been demonstrated that in the pathosystem of tomato with this phytoplasma, the pathogenesis might be phytoplasma-strain dependent, because two strains of ‘*Ca*. P. solani’ induced or repressed the genes associated with SA and JA metabolism and signaling, respectively (Ahmad and Eveillard, 2011).

Phytoplasmas depend on insect vectors for their transmission, and thus for their colonization of plants. On this basis, it has been suggested that these insect vectors can exploit phytoplasmas through independently evolved complex tritrophic interactions. The suppressed biosynthesis of JA via phytoplasma effector SAP11 and following disarmed plant defenses might promote insect vector reproduction on more susceptible plants (Kazan and Lyons, 2014; Tomkins et al., 2018). At the moment it is not known if JA repression during grapevine infection with the flavescence dorée phytoplasma is associated with any volatile organic compounds that might have attracted phytoplasma insect vectors, as has been demonstrated in apple tree infected with ‘*Ca*. P. mali’ with a SAP11-like effector (Janik et al., 2017). If this was the case, it might at least partially explain the epidemic nature of flavescence dorée disease, which in the presence of its vector *Scaphoideus titanus* spreads by a factor of 40 per year (Prezelj et al., 2013).

*Arabidopsis* plants that stably express an effector of a ‘*Ca*. P. asteris’ strain AY-OY, TENGU, have significantly lower levels of transcripts of genes that encode auxin response factors ARF6 and ARF8, which regulate floral development in a JA-dependent manner (Minato et al., 2014).

### Phytoplasma-Responsive Micro miRNA Expression Leads To Modulated Hormone Signaling

Some recent studies on phytoplasma-plant pathosystems have confirmed an important role of micro miRNAs in plant response to infection (Ehya et al., 2013; Gai et al., 2014a; Shao et al., 2016; Snyman et al., 2017; Chitarra et al., 2018). The analyses of phytoplasma-responsive miRNAs in grapevine, mulberry, Mexican lime, and Chinese jujube indicate that several miRNA are conserved among these plants. Moreover, miRNA may cooperatively modulate multiple hormone pathways and be involved in development of some symptoms observed in phytoplasma-infected plants.

Most published studies on phytoplasma-responsive miRNA suggest that miRNA-mediated auxin signaling regulates a response of plants to phytoplasmas. In particular, miR160 and miR166 that target for genes, which encode auxin response factors (ARF) from the family of transcription factors, were differentially expressed between uninfected and phytoplasma-infected Mexican lime (Ehya et al., 2013), mulberry (Gai et al., 2014a), Chinese jujube (Shao et al., 2016), and grapevine (Snyman et al., 2017). In addition, the same family of transcription factors was targeted with miR393 in infected Mexican lime and mulberry (Ehya et al., 2013; Gai et al., 2014a), and with miR164 in infected mulberry and Chinese jujube (Gai et al., 2014a; Shao et al., 2016). A down-regulation of miR159 was detected in phytoplasma infected Mexican lime (Ehya et al., 2013), Chinese jujube (Shao et al., 2016), and grapevine (Snyman et al., 2017). This microRNA targets for degradation of mRNAs that encode MYB transcription factors and have a role in plant hormone responses by redirecting auxin signal transduction by interacting with the carboxyl termini of ARFs (Zhang et al., 2011). The results of an analysis of phytoplasma–responsive microRNA in grapevine infected with flavescence dorée phytoplasma suggest that the alteration of JA biosynthetic pathway is promoted by tuning TCP by *TCP*/miR319 interplay through the downregulation of *JAZ3*, a target of both miR169 and the novel miC197-5p, and an ARF, target of miR167. Targets of miR167 were also ARFs in *Arabidopsis* infected with phytoplasma, as well as in TENGU-transgenic *Arabidopsis* plants (Minato et al., 2014). The system of *ARF*/miR167 influence both auxin signaling and JA pathway (Gutierrez et al., 2012; Boer et al., 2014) and it was suggested that the *ARF*/miR167 regulation together with detected decrease in JA concentration is the base of the flower alteration in infected plants (Chitarra et al., 2018). *In silico* analysis followed a detected differential expression of miR159 and miR319 in leaves of grapevine infected with ‘*Ca*. P. asteris’ and Chinese jujube infected with witches'-broom phytoplasma predicted that they target a transcriptional activator of gibberellin-dependent alpha-amylase (GAMYB)-like mRNA (Shao et al., 2016; Snyman et al., 2017), which may contribute to deformation of leaves. Regulation of the gibberellin regulatory network has also been predicted for miR167 during the potato infection with potato virus Y (Križnik et al., 2017). In has been shown in other systems that miR159, miR160, miR167, miR169, miR391, and miR393 are also involved in signaling pathway of abscisic acid (Sunkar et al., 2012), suggesting their vital role in plants subjected to stress conditions.

## Conclusions and Perspectives

The biology of phytoplasma and the responses of infected plants to their presence are still largely unknown due to their specific lifestyle, and the difficulties of their maintenance *in vitro* for performing controlled infections. However, a lot of new information has become available recently, which is supported by new transcriptomic proteomic and metabolomic data from phytoplasma-infected host plants. Moreover, with a development of different high-throughput techniques, applications of functional genomics technologies to model plants like *Arabidopsis* or *C. roseus*, and possibly with a routine cultivation of these intriguing bacteria, we are now at the starting point for eventually revealing the mechanisms, including the hormone crosstalk, by which phytoplasmas manipulate their hosts.

## Author Contributions

The author confirms being the sole contributor of this work and has approved it for publication.

### Conflict of Interest Statement

The author declares that the research was conducted in the absence of any commercial or financial relationships that could be construed as a potential conflict of interest.
